# Health economic evaluations of non-pharmacological interventions for persons with dementia and their informal caregivers: a systematic review

**DOI:** 10.1186/s12877-018-0751-1

**Published:** 2018-03-09

**Authors:** Franziska Nickel, Janina Barth, Peter L. Kolominsky-Rabas

**Affiliations:** 1 Interdisciplinary Centre for Health Technology Assessment (HTA) and Public Health (IZPH), Friedrich-Alexander-University Erlangen-Nürnberg (FAU), Schwabachanlage 6, 91054 Erlangen, Germany; 2 National Graduate College ‘Optimisation strategies in Dementia – OptiDem’, Karl and Veronica Carstens-Foundation, Essen, Germany

**Keywords:** Dementia, Non-pharmacological interventions, Economic evaluation, Costs, Cost-effectiveness

## Abstract

**Background:**

This systematic review aims to review the literature on trial-based economic evaluations of non-pharmacological interventions directly targeted at persons with dementia as well as persons with mild cognitive impairment and their respective caregivers.

**Methods:**

A systematic literature research was conducted for the timeframe from 2010 to 2016 in the following databases: Centre for Reviews and Dissemination, EconLit, Embase, Cochrane Library, PsycINFO and PubMed. Study quality was assessed according to the Drummond criteria.

**Results:**

In total sixteen publications were identified. Health economic evaluations indicated the cost-effectiveness of physical exercise interventions and occupational therapy. There was also evidence to suggest that psychological and behavioral therapies are cost-effective. Health economic studies investigating psychosocial interventions mainly targeted towards informal caregivers showed inconsistent results.

**Conclusions:**

Due to the increasing prevalence of dementia non-pharmacological interventions and their health economic impact are of increasing importance for health care decision-makers and HTA agencies.

**Electronic supplementary material:**

The online version of this article (10.1186/s12877-018-0751-1) contains supplementary material, which is available to authorized users.

## Background

Estimates show that there are around 47 million people living with dementia (PwD) worldwide [1]. In 2015, the global societal economic cost was estimated to be US$818 billion [1]. As the prevalence of dementia is projected to increase substantially, it poses significant challenges to health and social care systems [1].

Regarding treatment options, acetylcholinesterase inhibitors provide small but clinically important symptomatic benefits on cognition and function for persons with Alzheimer’s disease (AD), the most prevalent subtype of dementia, persons with Lewy body dementia and Parkinson’s disease dementia, without counteracting the progression of the disease [2–4]. Moreover, in persons with moderate-to-severe AD memantine has a small effect on cognition. However, these pharmacological interventions may provoke side-effects [4].

Furthermore, over the last decade non-pharmacological interventions for PwDs and persons with mild cognitive impairment (PwMCI) as well as their caregivers became more important, leading to a considerable growth in the evidence base [5, 6].

Results of meta-analyses indicate that physical exercise interventions may have a positive effect on the rate of cognitive decline in AD [7] and suggest that there is a beneficial effect on cognitive functioning independent of the subtype of dementia [8]. In contrast, a review of the Cochrane Collaboration [9] did not find clear evidence on the benefit of physical exercise programs on cognition of PwDs. The Cochrane meta-analysis showed that there may be a significant benefit from physical exercise interventions on the ability to perform activities of daily living in PwDs, but the rated quality of evidence was very low [9].

Further evidence suggests that psychological interventions can reduce symptoms of depression and anxiety among PwDs [10]. This is of particular importance, since depressive symptoms are common in all types and disease stages of dementia as well as in PwMCI and may impact considerably on the quality of life of PwDs and their caregivers [11]. With regard to caregiver interventions, a meta-analysis of high-quality RCTs shows that multicomponent interventions based on education and support elements delay the institutionalization of persons with AD, degenerative or mixed dementias [12].

Previously performed systematic reviews highlight the scarcity of economic evidence regarding non-pharmacological interventions for PwDs [13] and their supporting informal caregivers [14]. However, to inform resource allocation decisions, information on effective and cost-effective intervention strategies is essential for governmental decision-makers or payers [15].

Hence, the aim of this article is to conduct a systematic review of recent trial-based economic evaluations and cost studies of non-pharmacological interventions directly targeted at PwDs, PwMCI or their informal caregivers.

## Methods

### Search strategy

A systematic literature review was performed, searching the following key databases: Centre for Reviews and Dissemination (National Health Service Economic Evaluation Database; Health Technology Assessment), Cochrane Library, EconLit (EBSCO), Embase (OvidSP), PsycINFO (EBSCO) and PubMed (Medline). The timeframe for electronic searches was restricted to publications from 2010 to 2016. Key words concerning the dementia syndrome such as “dementia”, “Alzheimer’s Disease” etc. were combined with search terms in the context of economic evidence as, for instance, “cost”, “economic evidence”, “cost-utility” “cost-effectiveness” and “savings”. The complete research strategies for the respective databases are presented in the Additional file 1. Unpublished or grey literature was not included. The search was limited to studies in English or German.

### Study selection

Study eligibility was based on title and abstract screening, performed by two independent reviewers. A third independent reviewer was consulted in any case of disagreement. Articles passing the initial screening were retrieved for a detailed full text evaluation. The selection criteria are presented in Table 1.

**Table 1:** Inclusion and exclusion criteria

Inclusion criteria	Exclusion criteria
▪ Solely trial-based, non-modelling economic studies were included. ▪ The study design had to compare cost outcomes of non-pharmacological interventions directly targeted at persons with dementia, persons with mild cognitive impairment or the respective informal caregiver. ▪ The presence of an independent control group. ▪ No restriction was made regarding the type or stage of dementia. ▪ The studies had to be written in English or German, and published between 01.01.2010 and 31.12.2016.	▪ Model-based economic studies were excluded. ▪ Studies evaluating organizational changes and changes in the delivery of care and support were not considered. ▪ Qualitative research, cost-of-illness studies, case studies, studies without a control group, systematic reviews or meta-analyses were excluded. ▪ Conference abstracts were not considered.

This review includes studies based on standard economic evaluation methods. Cost-effectiveness analysis (CEA) measures and compares consequences in terms of an appropriate single natural effect or effect on a physical unit, which is common to all considered alternatives. The ratio of the mean incremental cost and the mean incremental effect of the interventions under evaluation is called incremental cost-effectiveness ratio (ICER). The ICER represents a summary measure that may support the decision making process by indicating the cost per unit change with respect to the evaluated outcome [15].

By contrast, cost-utility analysis (CUA) enables a comparison of multiple effects that are not necessarily common to the evaluated alternatives, as multi-dimensional health outcomes are captured into a single index [16]. In CUA health state preference scores (utility values) are used to value the states of health associated with the respective consequences of the interventions. Quality-adjusted life-years (QALY), which combine morbidity and mortality are the most frequently employed outcome measure in CUA [15].

### Data extraction

Data extraction was performed in line with the recommendation of the Centre for Reviews and Dissemination for reviews of economic evaluations [17], including type of economic evaluation, study objective, description of the intervention and comparators, measure of benefit, cost data and respective sources, methods for dealing with uncertainty as well as cost and outcome results.

### Quality appraisal

The appraisal of study quality was based on Drummond’s ten-item check-list for assessing economic evaluations [see Additional file 2] [15].

## Results

### Study selection

**Fig. 1:**
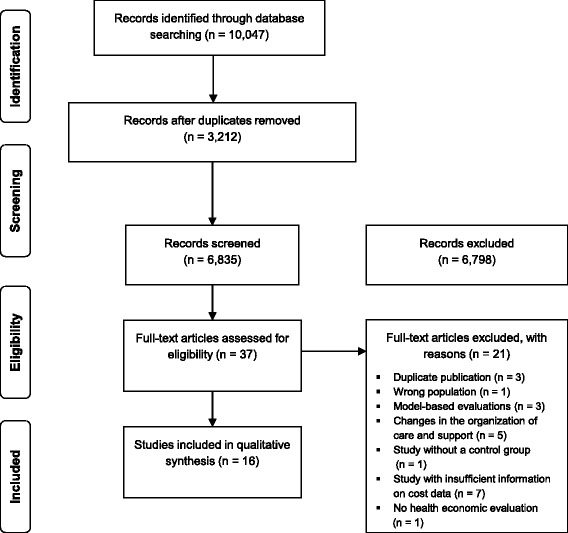
PRISMA flow chart

The systematic literature search identified 10,047 publications [see Additional file 1]. After the removal of duplicates, title screening of 6,835 articles was conducted. Subsequent to the screening of 390 abstracts, 37 articles were retrieved in order to assess the full texts. Of these, 16 were included in the synthesis. The flow of articles retrieved through electronic searches is depicted in Fig. 1.

### Basic characteristics of the included studies

The largest part of the included studies was conducted in the United Kingdom (seven studies). Of these, two articles are based on results from the British START (STrAtegies for RelaTives) study [18, 19]. Three studies were from the United States of America and two were from Finland. Further, one study each was conducted in Canada, the Netherlands, Denmark and Sweden.

Two articles report data on interventions that are mainly targeted at PwDs [20, 21], whereas seven studies comprise interventions, which involve both the PwD and the respective caregiver [22–28]. Six publications deal with programs that assist caregivers of PwDs [18, 19, 29–32]. Solely one study was targeted at PwMCI [33].

### Synthesis

The identified studies were grouped based on a classification proposed in the World Alzheimer Report 2011 [34].

Non-pharmacological interventions addressed to PwDs and, if applicable, to the patient-caregiver dyads were classified into the following categories:

i.) Physical exercise interventions;
ii.) Interventions to support and enhance cognitive abilities in PwDs as for instance reality orientation, reminiscence therapy or cognitive stimulation;
iii.) Psychological and behavioral therapies;
iv.) Occupational therapy.

Moreover, interventions that primarily target caregivers were classed together.

### Physical exercise interventions

Two trial-based CEA evaluating physical exercise interventions for PwDs [20, 28] and one for PwMCI were identified [33]. The exercise programs of the reviewed CEAs pursue different therapeutic goals, namely the reduction of behavioral and psychological symptoms of dementia (BPSD) [28] or the delay in the deterioration of patient’s physical functioning [20]. The intervention evaluated by Davis et al. [33] aims to improve executive cognitive functions in PwMCI. These different therapeutic goals are also reflected in the range of employed effectiveness measures (see Table 2).

**Table 2:** Characteristics of articles comprising physical exercise programs

Study, country	Type of study and economic evaluation	Time horizon C: Cost H: Health outcome	Study population, Number of participants	IG: Intervention group CG: Control group	Outcome measures (measures of benefit)	Cost data; source	Perspective	Results
D’Amico [28] UK, 2016	RCT CEA CUA	C: 12 weeks H: 12 weeks	Community-dwelling persons with a clinical diagnosis of dementia according to ICD-10 and one or more significant BPSD symptoms defined by the NPI as well as a caregiver willing to participate in the exercise training ▪ 131 randomized dyads ▪ 113 completed (89%) ▪ economic analysis subsample 52 dyads	IG: Dyadic exercise regimen (individually tailored walking program, for 20–30 min at least five times per week for 12 weeks, in the first 6 weeks the intervention was facilitated by a registered exercise professional) CG: CAU	Primary ▪ NPI Secondary ▪ ZBI ▪ GHQ ▪ DEMQOL-Proxy ▪ QALY	CSRI completed by the caregiver; intervention cost	(I) Health & social care perspective (II) Societal perspective	Potentially cost-effective considering behavioral and psychological symptoms, not cost-effective considering QALY gains. Mean cost difference between the groups were not significantly different over 12 weeks. This holds for both perspectives. From the societal perspective, the ICER was £421 per incremental difference in NPI score.
Davis [33] Canada, 2013	RCT CEA	C: 6 months H: 6 months	Community dwelling women aged 70 to 80 years; MMSE score ≥ 24; Subjective memory complaints, scored ≥6/8 on the Lawton and Brody Instrumental Activities of Daily Living scale ▪ 86 randomized participants	Three-trial arms: IG: Resistance training IG: Aerobic training CG: Balance and toning	▪ Executive cognitive function of selective attention and conflict resolution - Stroop test	Questionnaire to assess total healthcare resource utilization completed by the PwMCI	Health care system perspective	At 6 months resistance training and aerobic training yielded in health care cost savings when compared to controls doing balance and toning exercises.
Pitkälä [20] Finland, 2013	RCT CEA	C: 24 months H: 12 months	Community-dwelling persons with a clinical AD diagnosis and their spousal caregivers; 96% were receiving AD medication ▪ 210 randomized dyads ▪ Cost data 24 months: *n* = 210 ▪ Outcome assessed at 12 months: *n* = 161	Three-trial arms: IG: 1-year group-based exercise provided during visits to day care centers twice a week (1 h training) IG: 1-year tailored home-based exercise for 1 h twice a week administered by physiotherapists CG: CAU and subjects received oral and written advice on nutrition and exercise methods	▪ Patients physical functioning: FIM ▪ Mobility: SPPB	Data on use and cost of health and social services were retrieved from central registries and medical records of both patients and caregivers. Cost of patients and caregivers were summed.	Health and social care perspective (not explicitly stated)	Deterioration in patient’s physical functioning was delayed with the tailored home-based exercise program. Both intervention groups showed a significant reduction in the number of falls. The home-based exercise was found to be administered without increasing the total health and social service costs. The group exercise may even decrease the use and costs of health and social services.

*AD* = Alzheimer’s disease, *BPSD* = Behavioral and psychological symptoms of dementia, *CAU* = Care as usual, *CEA* = Cost-effectiveness analysis, *CSRI* = Client Service Receipt Inventory, *CUA* = Cost-utility analysis, *FIM* = Functional Independence Measure, *GHQ* = General Health Questionnaire, *ICD-10* = International statistical classification of diseases and related health problems, 10th revision, *ICER* = Incremental cost-effectiveness ratio, *PwMCI* = Person with mild cognitive impairment, *MMSE* = Mini-Mental State Examination, *NPI* = Neuropsychiatric Inventory; *QALY* = Quality-Adjusted Life Years, *RCT* = Randomized controlled trial, *SPPB* = Short Physical Performance Battery, *ZBI* = Zarit Burden Interview

In Finland a CEA was conducted concurrently with the Finnish Alzheimer disease exercise trial (FINALEX) [20]. In the first study arm a one-year group exercise program for community-dwelling persons with AD provided twice a week during visits to day care centers was assessed. The second arm included a one-year tailored home-based exercise program administered twice a week by physiotherapists. Both interventions were compared to a control group obtaining care as usual (CAU). To measure the impact of these intense and long term exercise programs on the physical functioning and mobility of the persons with AD, the Functional Independence Measure (FIM) and the Short Physical Performance Battery (SPPB) were used. Health outcomes were measured at baseline and after three, six and 12 months. Cost data was retrieved for the patient-caregiver dyads from central registries and medical records over a period of 24 months. Pitkälä and colleagues [20] came to the conclusion that an intensive and long-term exercise program administered in the patient’s home could slow the decline in physical functioning without increasing total health and social service costs.

A CEA conducted by D’Amico et al. [28] evaluated an intervention targeting community-dwelling PwDs who showed at least one BPSD. The intervention under evaluation was composed of a 12-week daily walking program (lasting for 20 to 30 min) performed by the patient-caregiver dyad in the surrounding of their residence. The dyads were instructed by a qualified exercise professional. The program was designed to become increasingly more intense, whereas the control group received CAU. The reduction in BPSDs was measured by means of the Neuropsychiatric Inventory (NPI) as the primary effectiveness outcome. Secondary analyses considered the impact on quality of life of the participants with dementia (measured using the DEMQOL-Proxy), caregiver burden (collected by employing the Zarit Caregiver Burden Interview (ZBI)) and caregivers’ mental health (measured using the General Health Questionnaire (GHQ)). Further, QALYs were estimated based on DEMQOL-Proxy scores. A subsample of 52 dyads was analyzed within the framework of the economic analyses [28]. In contrast to the entire sample [35], the economic subsample indicated a statistically significant between-group difference considering the average scores of the GHQ at 12 weeks, favoring the intervention group. The other evaluated outcomes did not show a significant difference at follow-up. The Client Service Receipt Inventory (CSRI) was completed by the caregiver in order to gather data on resource utilization. From the viewpoint of the health and social care system, the intervention group showed lower costs compared to the CAU control group, hence the intervention was dominant; however these results were not statistically significant. From a societal viewpoint, the ICER was estimated to amount to £421 per unit change in the NPI score. Assuming that a reduction of at least three points in the NPI score would be considered clinically relevant, the authors derived the suggestion that a clinically meaningful improvement could be achieved at a cost of £1,263. Therefore D’Amico et al. [28] concluded that the individually tailored walking program is potentially cost-effective when considering BPSD; however they also point to the fact that for the NPI no cost-effectiveness threshold was yet defined. The ICER for QALY was high, thus the intervention seems not to be cost-effective with regard to QALY gains.

In a Canadian three-arm trial, Davis et al. [33] assessed the cost-effectiveness of resistance training and aerobic training compared to a control group receiving balance and tone classes targeted at community dwelling women aged 70 to 80, who self-reportedly expressed to experience memory problems. As primary effectiveness outcome measure the executive cognitive function of selective attention and conflict resolution was assessed by means of the Stroop Test. From the point of view of the Canadian healthcare system, the incremental cost per incremental Stroop change score showed that aerobic training as well as resistance training decreased healthcare costs and proved to be more effective than twice weekly balance and tone classes. The study was limited by the fact that the participants did not have a formal mild cognitive impairment diagnosis. Moreover, the resource use data was collected through a self-completed questionnaire, which might have caused a recall bias.

### Interventions to support and enhance cognitive function

Three British RCTs were included that evaluated strategies to support and enhance cognition in PwDs [21, 26, 27]. The main considered outcomes were cognition and health-related quality of life (see Table 3).

**Table 3:** Characteristics of articles comprising cognitive interventions

Study, country	Type of study and economic evaluation	Time horizon C: Cost H: Health outcome	Study population, Number of participants	IG: Intervention group CG: Control group	Outcome measures (measures of benefit)	Cost data; source	Perspective	Results
Orgeta [27] UK, 2015	RCT CEA CUA	C: 26 weeks H: 26 weeks	Community-dwelling PwDs of any type (MMSE ≥10) and family caregivers ▪ 356 randomized dyads ▪ 273 dyads completed the second-follow-up (76.7%) ▪ 264 dyads with complete case information for the cost analyses	IG: Home-based individual cognitive stimulation therapy administered by the caregiver up to three times a week over 25 weeks CG: CAU	Primary* ▪ Cognition: ADAS-Cog (PwD) ▪ Self-reported QoL: QoL-AD (PwD) ▪ QALY (carer) derived from the EQ-5D-3 L Secondary PwD* ▪ Quality of caregiver-patient relationship: QCPR	CSRI	(I) Health & social care perspective (II) Societal perspective	CST did neither improve cognition or QoL of PwDs, nor carers’ physical and mental health. Costs of the intervention were offset by some reductions in social care and other services. Considering the primary outcomes for PwDs, it seems that the intervention is not more cost-effective compared to CAU (from both perspectives).
Woods [26] UK, 2012	RCT CEA CUA	C: 10 months H: 10 months	Community-dwelling PwDs with mild or moderate dementia and a respective caregiver willing to participate ▪ 488 randomized dyads ▪ 350 dyads completed the study	IG: Weekly reminiscence groups attended by both caregiver and patient over a period of 12 weeks followed by monthly maintenance sessions for a further 7 months CG: CAU	Primary (CEA) ▪ QoL-AD (PwD) ▪ GHQ of caregivers Secondary (CUA) ▪ EQ-5D of both caregiver and PwD ▪ EQ-5D proxy rated by the caregivers	CSRI	Public sector, multiagency perspective (NHS and local governments)	Joint reminiscence groups for PwDs and their caregivers are unlikely to be cost-effective. Potential beneficial effects for PwDs are offset by raised anxiety and stress in their caregivers.
D’Amico [21] UK, 2015	RCT CEA CUA	C: 6 months H: 6 months	Persons with mild-to-moderate dementia according to the DSM-IV criteria and a score between 0.5 and 2.0 on the Clinical Dementia Rating ▪ 236 randomized participants ▪ 199 completed the second follow up at 6 months	IG: After all participants completed 7 weeks of standard cognitive stimulation therapy (CST), the intervention group received MCST for 24 weeks in addition to usual care CG: 7 weeks of standard cognitive stimulation therapy (CST) and afterwards CAU	Primary ▪ ADAS-Cog ▪ QoL-AD Secondary ▪ MMSE ▪ ADCS-ADL ▪ NPI ▪ EQ-5D-3 L (PwD self-report or proxy) ▪ QoL-AD (proxy) ▪ DEMQOL	CSRI completed by caregivers or center workers	(I) Health & social care perspective (II) Societal perspective	Maintenance CST appeared cost-effective when looking at self-rated QoL and cognition (MMSE) and proxy-rated QoL as secondary outcomes. CST in combination with AChEIs offered cost-effectiveness gains when outcome was measured as cognition.

ADCS-ADL = Alzheimer’s Disease Cooperative Study-Activities of Daily Living Inventory, *AChEI* = Acetylcholinesterase inhibitor, *ADAS-Cog* = Alzheimer’s Disease Assessment Scale-Cognition subscale, *CAU* = Care as usual, *CEA* = Cost-effectiveness analysis, *CSRI* = Client Service Receipt Inventory, *CST* = Cognitive stimulation therapy, *CUA* = Cost-utility analysis, *DSM-IV* = Diagnostic and Statistical Manual of Mental Disorders, 4th Edition**,** *EQ-5D* = EuroQoL 5-dimensions, *GHQ* = General Health Questionnaire, *MCST* = Maintenance Cognitive Stimulation Therapy, *MMSE* = Mini-Mental State Examination, *PwD* = Person with dementia, *NHS* = National Health Service, *NPI* = Neuropsychiatric Inventory, *QCPR* = Quality of Caregiver-Patient Relationship**,** *QoL* = Quality of Life, *QoL-AD* = Quality of Life in Alzheimer’s Disease, *RCT* = Randomized controlled trial

*Only primary and secondary outcomes with significant between-group differences were presented in the original paper

Orgeta et al. [27] assessed structured, individual, home-based cognitive stimulation sessions for PwDs, administered by the respective caregiver. The caregivers were trained at home by researchers who also provided support via additional home visits or via telephone. Carers received a range of materials such as a manual, an activity workbook and a toolkit comprising boules and playing cards. The program was composed of 75 themed activity sessions. The sessions were designed to last 30 min and were scheduled to be delivered three times a week, over a period of 25 weeks. Compared to treatment as usual, the individual cognitive stimulation therapy (iCST) did not significantly improve cognition (measured by the Alzheimer’s Disease Assessment Scale-Cognition subscale (ADAS-Cog)) or quality of life of the PwD (assessed by the Quality of Life in Alzheimer’s Disease scale (QoL-AD)). This also applies for the caregivers’ physical and mental health (assessed by the Short Form questionnaire-12 items (SF-12)). Intervention costs were offset by some reductions in social care and other services. Even though improvements were neither observed with regard to most of the secondary outcomes for PwDs (activities of daily living, depression and BPSD), analyses indicated that PwDs in the intervention group may experience a higher relationship quality with their caregiver. From the health and social care perspective as well as from the societal perspective, there was no significant between-group difference with regard to mean costs. The authors concluded that considering the primary outcomes, from either perspective, the structured home-based cognitive stimulation does not seem to be more cost-effective than CAU. Further, the researchers remark that there are no available societal willingness-to-pay thresholds for the improvement in the primary outcomes measured by ADAS-Cog, QoL-AD or the Quality of Caregiver-Patient Relationship (QCPR) instrument. Considering the QALY gain for caregivers derived from the EQ-5D with societal weights, iCST was found to be more effective than CAU.

In another trial D’Amico et al. [21] evaluated the cost-effectiveness of a weekly maintenance cognitive stimulation therapy (MCST) over a period of 24 weeks compared to treatment as usual. Before entering the study, all participants completed seven weeks of twice-weekly standard cognitive stimulation therapy [36]. At six months, no difference between the control and intervention group was found with regard to the primary outcome cognition (measured by ADAS-Cog). However, the second primary outcome self-rated quality of life (assessed by means of the QoL-AD) was found to be higher in the intervention group compared to the CAU group. Participants receiving CAU showed only slightly non-significantly lower health and social care costs than participants in the MCST intervention group. The estimated ICER demonstrates that the mean cost for a 1 point difference on the QoL-AD scale was £266. The respective cost-effectiveness acceptability curve (CEAC) indicates that MCST can be considered cost-effective with a probability of 90% at a willingness-to-pay of around £1,400. In contrast, this probability was low considering cognition as outcome. Even though no significant between-group difference on the Mini-Mental Status Examination (MMSE) score was observed, the CEA indicated that MCST is likely to be cost-effective considering MMSE as outcome measure. Considering QALY gains derived from EQ-5D proxy-ratings, the mean incremental cost-utility ratio (ICUR) was £26,835. At a threshold of £20,000, CEAC suggests that considering the former outcome the probability of cost-effectiveness was 40%. Sensitivity analyses conducted from the societal perspective yielded inconsistent cost-effectiveness findings.

Within the framework of the REMCARE trial Woods et al. [26] evaluated the cost-effectiveness of weekly joint reminiscence groups provided for PwDs and their caregivers over a time span of 3 months, followed by monthly maintenance sessions over a period of 7 months. Primary outcomes were the PwD’s quality of life (measured by the QoL-AD) and carer’s mental health (assessed by the General Health Questionnaire-28 (GHQ-28)). With regard to these primary outcomes neither after 3 months nor after 10 months significant group-difference could be found. After 10 months, caregivers assigned to the intervention group even showed a significant increase in anxiety. Considering the mean health and social service costs for PwDs, the mean total costs for the intervention group was found to be 13.5% higher compared to the control group. However this between-group difference was not statistically significant. The estimated ICER for the QoL-AD was £2,586 (CI: -20,280 to 24,340). Woods et al. [26] concluded that the intervention was neither effective nor cost-effective.

### Psychological and behavioral interventions

Three publications evaluated psychological and behavioral therapies delivered to PwD-caregiver dyads (see Table 4) [22, 24, 25].

**Table 4:** Characteristics of articles on psychological and behavioral therapies

Study, country	Type of study and economic evaluation	Time horizon C: Cost H: Health outcome	Study population, Number of participants	IG: Intervention group CG: Control group	Outcome measures (measures of benefit)	Cost data; source	Perspective	Results
Spector [25] UK, 2015	RCT cost analysis	C: 6 months H: 6 months	Community-dwelling patients with mild-to-moderate dementia and anxiety (*N* = 50) ▪ 50 randomized participants ▪ 38 participants completed second follow-up assessment	IG: Ten-session cognitive-behavioral therapy for anxiety in dementia CG: CAU	Primary ▪ RAID Secondary ▪ CSDD ▪ QoL-AD (self-reported) ▪ NPI ▪ HADS ▪ MMSE ▪ QCPR	CSRI	Health and social care perspective	Significant improvements in depression. CBT found to be cost neutral.
Laakkonen [24] Finland, 2016	RCT CEA	C: 24 months H: 9 months	Community-dwelling PwDs (shortly after the diagnosis) and their spouses ▪ 136 randomized dyads ▪ 134 completed the follow-up assessment after 9 months	IG: Self-management group rehabilitation for PwDs and their spouses; enhancement of self-efficacy and problem solving skills CG: CAU and the study nurses gave participants in the control group oral and written advice on nutrition and exercise	Primary ▪ HRQoL: 15D (PwD) ▪ RAND-36 (caregiver) ▪ SCQ	Resource utilization of health and social services was retrieved from central registers and medical records	Health and social care perspective	Improvement of caregivers’ HRQoL and the cognitive function of the PwD without increasing total costs.
Søgaard [22] Denmark, 2014	RCT CUA	C: 3 years H: 3 years	Community-dwelling persons with a diagnosis of AD, mixed AD diagnosis and vascular disease or dementia with Lewy bodies within the past 12 months, MMSE ≥20 and a primary caregiver willing to participate ▪ 330 randomized dyads ▪ 195 dyads in the complete case analysis	IG: Intensive, multicomponent, semi-tailored psychosocial intervention program with counselling, education and support CG: The control group was informed about available support programs in their respective community	▪ Patient: EQ-5D proxy-rated by the caregiver ▪ Caregiver: self-rated EQ-5D	RUD and register data from national registries	Societal perspective	The psychosocial intervention is unlikely to be cost-effective since it did not generate additional QALY and it led to higher average usage of informal care. The provision of the intervention was estimated to incur an additional average cost of €3,401. Non-statistically significant cost savings were observed for the healthcare sector and for nursing home placements, whereas higher costs were observed for informal care.

*AD* = Alzheimer’s disease, *CAU* = Care as usual, *CBT* = Cognitive behavioral therapy, *CEA* = Cost-effectiveness analysis, *CSDD* = The Cornell Scale for Depression in Dementia, *CSRI* = Client Service Receipt Inventory, *CUA* = Cost-utility analysis, *EQ-5D* = EuroQoL 5-dimensions, *HADS* = Hospital Anxiety and Depression Scale, *HRQoL* = Health-related quality of life, *MMSE* = Mini-Mental State Examination, *PwD* = Person with dementia, *NPI* = Neuropsychiatric Inventory, *QALY* = Quality-Adjusted Life Year, *QoL-AD* = Quality of Life in Alzheimer’s Disease, *RAID* = Rating Anxiety in Dementia scale, *QCPR* = Quality of Caregiver-Patient Relationship, *RAND 36* = RAND 36-Item Health Survey, *RCT* = Randomized controlled trial; *RUD* = Resource Utilization in Dementia, SCQ = The Sense of Competence Questionnaire

A pilot RCT conducted by Spector et al. [25] in the UK assessed the cost of a ten-session cognitive-behavioral therapy (CBT) for patient-caregiver dyads compared to treatment as usual from the health and social care perspective. The Rating Anxiety in Dementia scale (RAID) was used as the primary outcome measure. The cost analysis yielded that at 15 weeks the adjusted difference in anxiety of the PwDs was lower in the CBT group compared to the control group ( -3.10; 95% CI: -6.55 to 0.34). This finding was maintained at 6 months ( -4.59; 95% CI: -9.34 to 0.15). Further, at 15 weeks depression of the PwDs measured by the adjusted Cornell Scale for Depression in Dementia (CSDD) was lower in the CBT group ( -5.37; 95% CI: -9.50 to -1.25), which remained significant at 6 months. The total cost that occurred over the 6-month follow-up period were found to be significantly lower within the CBT group compared to CAU (adjusted mean difference -£564.38 (95% CI: -£1,252.08 to -£112.85)). While also including intervention cost, estimated to amount to £1,002 per person, the total cost from the health and social service perspective were lower for the control group, with an adjusted mean difference of £769.80 (95% CI: -£121.99 to £1,697.38). However, this difference was not found to be statistically significant. Hence, the authors concluded that CBT proved to be cost neutral.

A Finnish trial conducted by Laakkonen et al. [24] performed a CEA, evaluating a self-management group rehabilitation program for PwDs and their spouses administered shortly after the diagnosis. The intervention comprised eight group sessions of self-management offered to the spousal dyads and was compared to CAU. At 3-month, the intervention group demonstrated higher HRQoL outcomes as measured with the Finnish version of the RAND 36-Item Health Survey in spouses of PwD, however after 9 months the effect was weakened. With respect to spouses’ sense of competence and feelings of mastery (assessed by the Sense of Competence Questionnaire (SCQ)) no significant group difference was demonstrated. Further, cognitive scores of PwDs in the intervention group improved significantly more than those of the control group. These positive effects were not accompanied with higher health and social services costs; hence the authors concluded that the self-management group rehabilitation is cost neutral.

As part of the Danish Alzheimer’s Intervention Study, which assessed the provision of an early psychosocial intervention for community-dwelling PwDs with a recent diagnosis and their respective caregivers, a CUA was conducted from the societal perspective. Søgaard and his colleagues [22] evaluated an intensive, multicomponent, semi-tailored psychosocial intervention program over a period of 3 years. The program was composed of counselling, educational and support elements, which were compared to a control group that was informed about available support programs in their respective communities [37]. QALY were estimated individually for the PwD and the respective caregiver and eventually aggregated for the analysis. HRQoL values of the PwDs were measured by means of proxy-rated EQ-5D. Cost data on informal care and production loss was collected by means of the Resource Utilization in Dementia (RUD) instrument. Data on resource utilization in the primary and secondary health sector was based on national registers. Within both study arms, no significant difference was observed between QALYs and cost measures. The observed cost increase with respect to informal care was not outweighed by the savings in the formal care sector [22].

### Occupational therapy

Gitlin et al. [23] examined the cost-effectiveness of a structured intervention administered by an occupational therapist composed of six in-home sessions and two telephone contacts over a period of 4 months (see Table 5). The intervention was structured into 3 parts: firstly the capabilities of the PwD were assessed. Secondly, an identification of activities that are tailored to the prevailing capabilities of the PwD was conducted and caregivers were instructed on support strategies. Eventually, when the activities were mastered, the occupational therapist was giving advice on how to employ the practiced techniques to other care challenges. The intervention reduced the time the caregiver was occupied by activities provided in relation to the PwD and was found to be cost-effective compared to treatment as usual.

**Table 5:** Characteristics of a trial on occupational therapy

Study, country	Type of study and economic evaluation	Time horizon C: Cost H: Health outcome	Study population, Number of participants	IG: Intervention group CG: Control group	Outcome measures (measures of benefit)	Cost data; source	Perspective	Results
Gitlin [23] US, 2010	RCT CEA	C: 4 months; H: 4 months	Caregivers of PwDs with a MMSE score < 24, mild to moderate dementia and showed at least one behavioral symptom; Caregivers living with the PwD, providing at least 4 h of daily care ▪ 60 randomized dyads	IG: Tailored home-based activity program delivered by occupational therapists with 8 sessions of occupational therapy over 4-month consisting of the assessment of patient’s abilities, caregiver communication & home environment. Development of three activities tailored to patient capabilities. CG: Wait-list control group that did not receive any study-related contact.	Two items from the Caregiver Vigilance Scale: (1) number of hours per day the caregiver is actually doing things for the PwD (2) number of hours per day the caregiver feels the need to be there or on duty to care for the PwD	Total average intervention cost	Perspective of the individual caregiver	Intervention caregivers saved: (1) one extra hour per day doing things for the PwD at a cost of $2.37/day (2) one extra hour per day feeling the need to be there or on duty to care for the PwD at a cost of $1.10/day.

*CEA* = Cost-effectiveness analysis, *MMSE* = Mini-Mental State Examination, *PwD* = Person with dementia; *RCT* = Randomized controlled trial

### Psychosocial interventions mainly targeted at the caregiver

Six publications were found that focused on five interventions mainly delivered to informal caregivers of PwDs (see Table 6) [18, 19, 29–32].

**Table 6:** Characteristics of articles comprising psychosocial interventions mainly targeted at the caregiver

Study, country	Type of study and economic evaluation	Time horizon C: Cost H: Health outcome	Study population, Number of participants	IG: Intervention group CG: Control group	Outcome measures (measures of benefit)	Cost data; source	Perspective	Results
Joling [29] Netherlands, 2013	RCT CEA CUA	C: 12 months H: 12 months	Caregivers of community-dwelling PwD and at least one other family member or friend available to take part in the family meetings ▪ 192 randomized caregivers ▪ 87 participants with complete cost and primary outcome data at the follow-up assessment at 12 months	IG: Family meetings intervention with six in-person counselling sessions led by trained counselors with a background in a health care related profession. The family meetings aimed to provide psycho-education, problem solving techniques and to mobilize the existing family networks. CG: CAU	▪ MINI caregiver ▪ PwD: proxy-rated SF-12 ▪ Caregiver: SF-12 ▪ PwD QALY ▪ Caregiver QALY ▪ PwD-caregiver dyad QALY	▪ Cost diaries and interview assessments to measure informal care time ▪ Direct and indirect costs of both the caregiver and patient were gathered.	Societal perspective	Family meetings cannot be considered as cost-effective compared to CAU. No significant differences in total costs between both groups were observed. Ambulatory care costs among caregivers in the intervention group were higher compared to the CAU group. No between groups differences in QALY for both patients and caregivers or on clinical mental parameters for caregivers were found.
Dahlrup [30], Sweden, 2013	Quasi-experimental cohort study, CUA	C: up to 60 months H: up to 60 months	Family caregivers of recently diagnosed community-dwelling PwDs ▪ 308 randomized participants	IG: Psychosocial intervention composed of education and provision of a support group for family caregivers of PwDs (5 weekly counselling sessions, followed by a group discussion). Caregivers who were unable to join group sessions received individual meetings. CG: CAU	▪ Caregivers HRQoL: EQ-5D (two subsets were considered: PwDs still residing in the community and PwDs who had moved to a nursing home)	Municipality registers provided data on resource use in terms of home help service and cost of formal care for PwDs and caregivers	Not explicitly stated.	No group difference in cost was found. Significant higher HRQoL for caregiving children and grandchildren receiving the intervention.
Wray [31] US, 2010	RCT cost analysis	C: 12 months	Caregivers of community-dwelling veterans with at least a moderate level of dementia who are dependent on their respective caregiver in performing ADL and IADL. Caregivers were spousals, experiencing at least a moderate level of caregiver strain ▪ 158 randomized caregiver-veteran dyads	IG: 10-week telephone support group for caregivers of veterans with dementia. The Telehealth Education Program is composed of caregiver education and support CG: CAU	Not stated.	Health care utilization and cost data derived from the veterans Information System Technology Architecture databases. Data included inpatient, nursing home, outpatient and outpatient pharmacy files.	Not explicitly stated.	The telephone support groups yielded in significant short term average cost savings of $2,768 per patient at 6 months as compared with CAU, however these were not maintained at 12-month.
Van Houtven [32] US, 2012	RCT cost analysis	C: 12 months O: 12 months	Caregivers of person’s with AD or Parkinson’s disease ▪ 187 randomized dyads	IG: Caregiver skill training over a 24-week period. In-home or telephone-based sessions delivered by a trained nurse CG: Over the same 24-week period the wait-list comparison got social phone contacts administered by persons trained for the provision of socially supportive conversations by phone	Not reported.	Out-of-pocket costs of caregivers assessed by the caregiving assistance measure (including service use of the care recipient & the caregiver himself)	Individual caregiver perspective	Caregiver skill training yielded in clinically and statistically significant reduction in caregivers’ depression and improved caregiving mastery. It increased the likelihood of caregivers to experience any out-of-pocket expenditure (OOP) by 26% over usual care; overall OOP costs were not significantly altered.
Livingston [19] UK, 2014	RCT CEA CUA	Short-term C: 8 months H: 8 months Long-term C: 24 months H: 24 months	Family caregivers providing support at least once a week to community-dwelling PwD ▪ 260 randomized dyads	IG: Manual-based individual coping intervention for caregivers delivered in 8 sessions by supervised psychology graduates CG: CAU	Primary ▪ Carer: HADS-T Secondary ▪ Carer: QALY derived from the EQ-5D and societal weights	CSRI	Health and social care perspective	The intervention was clinically effective in terms of carers’ anxiety and depression as well as their quality of life. Moreover it was found to be cost-effective in the short and long term when considering caregiver-related cost.
Livingston [18] UK, 2014	RCT CEA CUA	Short-term C: 8 months H: 8 months Long-term C: 24 months H: 24 months	Family caregivers providing support at least once a week to community-dwelling PwD ▪ 260 randomized dyads ▪ 227 dyads completed 8 months follow-up session ▪ 196 randomized dyads completed 24 months follow-up session	IG: Manual-based individual coping intervention for caregivers delivered in 8 sessions by supervised psychology graduates CG: CAU	Carer: HADS-T Carer: QALY derived from the EQ-5D and societal weights Patient: QoL-AD (proxy-rated)	CSRI	Health and social care perspective	The intervention was clinically effective and cost-effective in the short and long term when considering carer plus PwD costs.

*AD* = Alzheimer’s disease, *ADL* = Activities of Daily Living, *CAU* = Care as usual, *CEA* = Cost-effectiveness analysis, *CSRI* = Client Service Receipt Inventory, *CUA* = Cost-utility analysis, *EQ-5D* = EuroQoL 5-dimensions, *HADS-T* = Hospital Anxiety and Depression Scale (total score), *HRQoL* = Health-related quality of life, *IADL* = Instrumental Activities of Daily Living, *MINI* = Mini International Neuropsychiatric Interview, *PwD* = Person with dementia, *QALY* = Quality-Adjusted Life Year, *QoL-AD* = Quality of Life in Alzheimer’s Disease, *RCT* = Randomized controlled trial, *SF-12* = 12-Item Short Form Health Survey

A Dutch CEA and CUA conducted by Joling et al. [29] evaluated the effectiveness of a family meetings intervention on caregiver’s mental health compared to CAU from the societal perspective. Data was gathered alongside a RCT of 192 primary caregivers of community-dwelling PwD. The intervention consisted of two individual meetings between the caregiver and a trained counsellor and four structured family meetings with additional relatives and friends joining in. Apart from the scheduled sessions, the counsellor was available for support via telephone. The objective of the family meetings was to provide psychoeducation, problem solving techniques and to mobilize the existing family networks. However, after 12 months, significant effects on caregiver’s depression and anxiety could not be observed. The same applies for caregiver’s and patient’s quality of life. Further, over the 12 months, no significant cost difference between the intervention and control group was measured. Total cost per patient-caregiver dyad was estimated to be EUR 77,832 in the family meetings group compared to EUR 75,201 in the CAU group (mean adjusted difference EUR 4,149; 95% CI: -13,371 to 21,956; ICER 157,534). Hence, the researchers drew the conclusion that compared to CAU the provision of family meetings cannot be considered a cost-effective strategy.

A Swedish CUA by Dahlrup et al. [30] evaluated the cost-effectiveness of a psychosocial intervention for family caregivers of PwDs including five weekly counselling sessions administered by a registered nurse and a counselor. Education and information on dementia as well as available services in the community were followed by a group discussion. Caregivers who were unable to join group sessions received individual meetings and were invited to continue the support groups twice a month over a period of three months. 12 months after the fifth educational session, a follow-up was offered for each group. Over the time span of the study, caregivers in the intervention group were given the opportunity to contact a physician, the administering nurse or the counselor. With regard to the endpoints survival and time to institutionalization of the PwD, the intervention group did not differ significantly from the control group with CAU. A subgroup analysis based on the relation between caregiver and PwD was conducted, which demonstrated that if the caregiver was a spouse or cohabitant, PwDs transition into the nursing home occurred earlier compared to the control group (*p* < 0.01). In case the caregiver was a child or grandchild of a PwD, patients in the intervention group stayed longer in the community compared to controls (*p* = 0.06). The analysis of caregiver’s Health Related Quality of Life (HRQoL) was distinguished into caregivers whose care recipient was still living in the community and PwDs that had moved to a nursing home. For the former subgroup, caregiver’s health-related quality of life measured by EQ-5D was significantly higher in the intervention group compared to the controls. There was no significant in between-group difference with regard to caregivers whose care recipient moved to a nursing home. Depending on the caregiver’s relation to the care recipient, HRQoL differed. Resource use for home help services, nursing home care and the study intervention were collected. The cost analysis accounted for the differing length of observation. Over the 5-year follow-up, the median total cost weighted for study length did not differ between the two groups under consideration. For the intervention group the median total cost per month amounted to EUR 1,926 (interquartile range 1,043 to 2,588) in comparison to EUR 1,860 (interquartile range 864 to 2,577) in the control group (*p* = 0.47). Hence, it can be concluded that the intervention yielded significantly higher HRQoL in children and grandchildren caring for a PwD without significantly increasing the evaluated cost (i.e. costs for home help services, nursing home placement and the intervention cost).

An American cost analysis performed by van Houtven et al. [32] evaluated the effect of skill training aiming to reduce depressive symptoms of family caregivers of persons with AD or Parkinson’s disease on the caregiver’s out-of-pocket (OOP) costs. The skill training lasted over 24 weeks and included in-home or telephone-based sessions administered by a trained nurse. The control group was composed of the wait-list participants, who received supportive consultations via phone. Van Houtven and colleagues [32] reported clinically and statistically significant reductions in depressive symptoms and positive effects on caregiving mastery for the participants in the intervention group. The cost analysis solely included OOP costs faced by the caregiver for services provided to the care recipient or the caregiver himself. The analysis indicates that the intervention increased the likelihood of caregiver’s OOP expenditure by 26% points, however no intervention effect was observed with regard to the overall level of expenditures. It was acknowledged by the authors that OOP cost form only a part of the total economic cost which caregivers may face [32].

Wray et al. [31] examined the effect of the provision of a telehealth education program to spousals of veterans with dementia in the United States of America. The telephone support group included educational aspects on the syndrome as well as training on emotion-focused and problem-focused coping strategies and was administered over a period of ten weeks. Health care utilization and cost data were retrieved from the veterans’ databases. The cost analyses showed that in the short-term, 6 months after the intervention start, the total health care costs were significantly lower in the intervention arm in comparison to the CAU control (*p* = 0.039). The same applies when separately considering nursing home cost (*p* = 0.009). Mean overall cost savings achieved in the intervention group amounted to $2,768 per PwD. Of these, mean nursing home cost savings per PwD were $1,059. However, over the 12-month follow-up, the decrease in total care cost was not maintained.

Livingston and colleagues [19] evaluated the short and long-term cost-effectiveness of a manual-based individual coping strategy intervention (START) targeted at family caregivers of PwD in the UK. The manual was based on the Coping with Caregiving intervention developed in the US. The START intervention consisted of eight psychological therapy sessions, administered by psychological graduates. The sessions covered psychoeducation about dementia, caregiver stress and behavior strategies [38]. The short-term analysis over a period of 8 months with QALYs as outcome measure yielded that caregivers receiving the intervention showed higher, however non-significant, health and social care costs amounting to £252 (95% CI: £28 to £565) compared to CAU and an incremental QALY gain of 0.042 (95% CI: 0.015 to 0.071). With the total score of the Hospital Anxiety and Depression Scale (HADS-T) as outcome the incremental health and social care costs were estimated to be £247 (95% CI: £0 to £569) higher in the START group. The ICUR was £6,000 per additional QALY and £118 per unit change on HADS-T. The likelihood that the START intervention was cost-effective at a willingness-to-pay threshold of £20,000 was 93%. In the long-term (after 24 month) for the QALY analysis, the cost per additional QALY was £11,200 and the CEAC indicated that the likelihood that the intervention was cost-effective was 75% presuming a willingness-to-pay threshold of £30,000. The authors concluded that the START intervention was cost-effective over 8 and 24 months.

A second publication of Livingston et al. [18] additionally assessed PwD’s outcomes and costs. The analyses showed that when combining carer and PwD’s costs, the START intervention also dominates CAU considering caregiver’s outcomes. However, with regard to PwDs quality of life measured by means of the QoL-AD, no significant between-group difference was found.

### Assessment of study quality according to the Drummond criteria

Considering the methodological quality of the included studies, it can be acknowledged that the majority of articles gave a detailed description of the considered alternatives [see Additional file 2]. In most studies a comparison was made between a non-pharmacological intervention and CAU, whereas Davis et al. used a popular and widely available exercise program in order to reflect the actual practice in the respective community [33]. Two publications did not present detailed effectiveness results in the publication and the referenced articles could not be found [31, 32].

#### Perspective

Three studies did not explicitly state the employed perspective [20, 30, 31]. Considering the articles which stated their perspective, the most prevalent was the health and social care perspective, which is not taking into account informal care costs. Three studies reported both data from the health and social care perspective as well as from the societal perspective [21, 27, 28]. Nonetheless, the majority of identified studies also incorporated health outcomes of informal caregivers. Independent of the employed perspective some identified evaluations summed up the health and social care cost of both caregiver and PwD in order to reflect the effect on caregiver burden [20].

#### Time horizon

The time horizon of the economic analyses ranged from 3 up to 60 months. Considering the continuous progression of most types of dementia, data on long-term cost-effectiveness would be important. Therefore, short-term analyses might not be able to capture the full consequences on health outcomes and resource utilization.

## Discussion

This systematic review highlights recent evidence on health economic evaluations of non-pharmacological interventions for PwDs or PwMCI and their respective informal caregivers. To our knowledge this is the first systematic review focusing on psychosocial interventions directly delivered to patients or informal caregivers. Additionally, the methodological quality of the included health economic studies was evaluated according to the criteria by Drummond et al. [15].

In the light of the comparably low success rate in the development of AD drugs [39] and the limited number of agents in the current pipeline [40], the development of effective non-pharmacological interventions is of particular importance in the treatment and care of PwDs and the support of informal caregivers. This is also reflected in the increasing research conducted in this field [5] and the establishment of the pan-European research network INTERDEM aiming to promote collaboration and research on psychosocial interventions [6].

A previous systematic review detected a substantial lack of economic evidence on non-pharmacological interventions for PwDs [13]. In particular no economic evidence on physical exercise interventions could be identified by Knapp et al. [13].

In the meantime the evidence base has been slightly growing. The three identified studies concerned with exercise interventions showed that the programs were found to be potentially cost-effective for specific outcomes. An individually tailored home-based exercise program significantly delayed the deterioration of physical functioning of AD patients [20]. Further, an instructed walking program for PwDs and their caregivers is potentially cost-effective compared to CAU, when focusing on the reduction of BPSD as the outcome of interest [28]. However, the underlying effectiveness evaluation based on a larger sample did not prove the effectiveness with regard to this outcome [35]. Therefore, further evaluations are needed.

A structured occupational therapy intervention was found to be cost-effective from the caregiver’s perspective, as it led to a reduction in caregiving time [23]. A previous CEA on occupational therapy also demonstrated cost-effectiveness from the societal perspective [41].

Considering interventions to support and enhance cognition of PwDs, the evidence identified within this review is inconsistent. Joint reminiscence groups for PwDs and caregivers are found unlikely to be cost-effective compared to CAU [26]. The same findings were made for a carer-led CST intervention [27]. In contrast, a previous study conducted by Knapp et al. [42] found CST to be potentially cost-effective in comparison to CAU, considering its effects on cognition and quality of life. Likewise our systematic review found evidence that a long-term maintenance CST is potentially cost-effective [21].

With regard to psychological and behavioral treatments, self-management group rehabilitation for PwDs and their spouses [24] as well as cognitive-behavioral therapy for PwD-caregiver dyads [25], demonstrated effectiveness and cost-neutrality. In contrast, an intensive, multicomponent, semi-tailored psychosocial intervention program with counselling, education and support program was not found to be cost effective from the societal perspective [22].

Another previous systematic review by Jones et al. [14] focused on economic evaluations assessing interventions targeted at informal caregivers of community-dwelling PwDs. The authors indicated a lack of evidence and the need to gather carer data alongside patient data. In the framework of this previous review, a social support intervention comprising a facilitated access to a befriender volunteer was unlikely to be cost-effective [43]. Moreover, a home or telephone-based problem-solving therapy improved coping skills for a subgroup of caregivers without a significant difference in caregiver expenditures [44]. The evidence with regard to interventions directed at informal caregivers identified within this systematic review was also mixed. A manual-based individual coping program for informal caregivers was found to be cost-effective in the short and long term [18, 19]. Further, an educational and support intervention appears to be effective and cost neutral [30]. In contrast, a family meetings intervention is not considered to be cost-effective compared to CAU [29].

### Methodological challenges of economic evaluations of dementia populations

Regarding the methods of economic evaluations, the researchers of the identified trials most frequently applied CEA. As cost effectiveness measures, for instance, the incremental cost per one-point difference in NPI score [28] or per QoL-AD score [18, 21] were used. However, no cost-effectiveness thresholds exist for outcomes such as the change in the NPI or the MMSE score [45].

Moreover, the assessment of HRQoL in PwDs may be prone to recall bias and missing values are likely to occur within a cognitively impaired population [46]. Regarding proxy measures, a Dutch study conducted by Arons et al. indicates that patient-by-proxy HRQoL values must be interpreted cautiously, as caregivers are found to reflect parts of their own HRQoL onto the care recipient [47].

The CUAs that were included in this systematic review derived QALY of the PwD from dementia-specific instruments such as the DEMQOL and DEMQOL Proxy. Besides, generic proxy-rated caregiver EQ-5D values were employed to assess PwD’s HRQoL. Concerning the EQ-5D, a study of Aguirre and colleagues [48] suggests that the EQ-5D may not capture all relevant aspects associated with dementia and PwD’s experiences. However, Aguirre et al. [48] showed that compared to the dementia-specific measures DEMQOL and the Qol-AD, the EQ-5D demonstrated adequate psychometric properties and good reliability within this population. In addition, Orgeta et al. [49] indicated that the EQ-5D enables PwDs in mild and moderate stages to rate their own HRQoL. However, the study found significant difference between caregiver-rated and self-rated HRQoL. The ratings were influenced by the type of the caregiving relationship. Children of PwDs rated the HRQoL lower in comparison to spousal caregivers. Therefore, the authors recommend to use both self and proxy ratings in economic evaluations of interventions for PwDs [49].

In contrast, Algar and colleagues [50] propose observational measures for the effectiveness assessment of non-pharmacological interventions for PwDs. Especially, in more severe levels of dementia observational methods may provide additional valuable data and might be an alternative way to capture PwD’s experiences [50]. The use of observational tools in health economic analyses might therefore be a valuable field of research.

There are as well challenges arising with regard to the identification and valuation of costs. A systematic review on cost-of-illness studies showed that the main cost drivers in the dementia care context are informal care costs [51]. Moreover, Wimo et al. [52] point to the complex interaction between those who may benefit from the consequences of the interventions and those who finance the care. The societal perspective includes all relevant cost irrespective where they occur and where they are financed. Therefore, Wimo and colleagues [52] recommend that taking on a societal perspective is often preferable.

Another systematic review by Krol et al. [53] assessed the effect of the inclusion and exclusion of informal care cost and effects on cost-effectiveness outcomes. The authors concluded that the inclusion of informal care can have a considerable impact on cost-effectiveness outcomes [53]. Moreover, Shearer et al. [54] recommend that the methods employed to measure and value informal care should be explicitly stated. In addition, sensitivity analyses should be employed to show the variability of the results using alternative methods [54].

### Limitations

Even though a comprehensive literature search based on broad search terms was conducted, some papers meeting the search criteria might not have been identified. Moreover, this systematic review may be subject to a language bias, as solely publications in English and German were included.

A narrative synthesis was conducted due to the heterogeneity of the identified studies with regard to the employed health outcomes, perspectives of the analyses and time horizons. Furthermore, the study populations under examination comprised different types and severity levels of dementia. Moreover, the generalizability of the findings is limited since the identified studies were conducted in North American and European countries, with different underlying health and social care systems.

### Implications for future research

There is evidence on the effectiveness of multicomponent psychosocial interventions on the maintenance of caregivers’ psychological health [55] and delayed institutionalization of PwDs [12]. Furthermore, a recent study by Straubmeier et al. [56] shows a highly promising effect of a structured multicomponent intervention targeted at PwMCI and persons with mild to moderate dementia on their cognitive abilities and activities of daily living [56]. Future research should therefore examine the cost-effectiveness of multicomponent interventions and consider subgroups of PwDs at different disease stages. Moreover, the homogeneity of the respective caregivers should be taken into account.

Selected non-pharmacological interventions are recommended by most guidelines. However, there is still a lack of information on their dissemination as well as on facilitators and barriers of the translation of evidence on non-pharmacological interventions into practice.

## Conclusions

This review provides evidence on economic aspects of non-pharmacological interventions in the therapeutic field of dementia. Health economic evaluations suggest that exercise programs, occupational therapy as well as cognitive and psychological interventions directly delivered to PwDs demonstrate cost-effectiveness compared to CAU with regard to specific outcomes. No economic evaluations on sensory interventions and creative interventions such as art, music or dance therapy could be identified.

## Additional files

Additional file 1: Search strategies and search results. (PDF 106 kb)

Additional file 2: Quality assessment of included studies according to the 10-item Drummond check-list. (PDF 199 kb)

## Abbreviations

AChEI: Acetylcholinesterase inhibitor
AD: Alzheimer’s disease
ADAS-Cog: Alzheimer’s Disease Assessment Scale-Cognition Subscale
ADCS-ADL: Alzheimer’s Disease Cooperative Study-Activities of Daily Living Inventory
BPSD: Behavioral and psychological symptom of dementia
CAU: Care as usual
CBT: Cognitive-behavioral therapy
CEA: Cost-effectiveness analysis
CEAC: Cost-effectiveness acceptability curve
CI: Confidence Interval
CSDD: Cornell Scale for Depression in Dementia
CSRI: Client Service Receipt Inventory
CST: Cognitive stimulation therapy
CUA: Cost-utility analysis
DSM-IV: Diagnostic and Statistical Manual of Mental Disorders, 4th Edition
EQ-5D: EuroQoL 5-dimensions
FIM: Functional Independence Measure
FINALEX: Finnish Alzheimer disease exercise trial
GHQ: General Health Questionnaire
HADS: Hospital Anxiety and Depression Scale
HADS-T: Hospital Anxiety and Depression Scale (total score)
HRQoL: Health-Related Quality of Life
IADL: Instrumental Activities of Daily Living
ICD-10: International statistical classification of diseases and related health problems, 10th revision
ICER: Incremental cost-effectiveness ratio
iCST: Individual cognitive stimulation therapy
ICUR: Incremental cost-utility ratio
MCST: Maintenance cognitive stimulation therapy
MINI: Mini International Neuropsychiatric Interview
MMSE: Mini-Mental Status Examination
NHS: National Health Service
NPI: Neuropsychiatric Inventory
OOP: Out-of-pocket
PwD: Person with dementia
PwMCI: Person with mild cognitive impairment
QALY: Quality-adjusted life-years
QCPR: Quality of Caregiver - Patient Relationship
QoL: Quality of Life
QoL-AD: Quality of Life in Alzheimer’s Disease scale
RAID: Rating Anxiety in Dementia scale
RAND 36: RAND 36-Item Health Survey
RCT: Randomized controlled trial
RUD: Resource Utilization in Dementia
SCQ: Sense of Competence Questionnaire
SF-12: Short Form questionnaire-12 items
SPPB: Short Physical Performance Battery
ZBI: Zarit Caregiver Burden Interview
