# Supplementary material for: Health economic evaluations of non-pharmacological interventions for persons with dementia and their informal caregivers: a systematic review
Source: BMC Geriatr. 2018 Mar 9;18:69. doi: 10.1186/s12877-018-0751-1 (PMC5845149; doi:10.1186/s12877-018-0751-1)
Supplement: Supplementary file 1 — Search strategies and search results. (PDF 106 kb) [file 12877_2018_751_MOESM1_ESM.pdf]

Additional file 1: Search strategies and search results

Search strategies

EMBASE

(Cost or costs or economic evidence or cost-utility or cost-effectiveness or cost-benefit or savings or "Cost Analysis" or "Economics" or "Cost Savings" or "Cost-Benefit Analysis" or "Health Care Costs") and (Dementia or "Frontotemporal Dementia" or Vascular Dementia or Alzheimer Disease or Alzheimer's or Alzheimer's Disease or Lewy Body Disease or MCI or "Mild cognitive impairment").af.

Limit limit to yr="01.01.2010-31.12.2016"

Cochrane Library

(dementia or "frontotemporal dementia" or Alzheimer or "Lewy Body Disease" or MCI or "mild cognitive impairment" or "vascular dementia" or "Alzheimer's Disease") and (Cost or costs or economic evidence or cost-utility or cost-effectiveness or cost-benefit or saving)

Publication Year 01.01.2010-31.12.2016 (Word variations have been searched)

PsycINFO

TX (dementia OR frontotemporal dementia OR Alzheimer Disease OR "Lewy Body Disease" OR MCI OR "mild cognitive impairment" OR vascular dementia OR Alzheimer's Disease) AND (TX (Cost OR costs OR economic evidence OR cost-utility OR cost-effectiveness OR cost-benefit OR saving))

Limiters - Publication Year: 01.01.2010-31.12.2016; Publication Type: All Journals

Expanders - Apply equivalent subjects; Apply related words; Also search within the full text of the articles; Search modes - Boolean/Phrase

PubMed

(Cost OR costs OR economic evidence OR cost-utility OR cost-effectiveness OR cost-benefit OR savings OR "Costs and Cost Analysis"[Mesh] OR "Economics"[Mesh] OR "Cost Savings"[Mesh] OR "Cost-Benefit Analysis"[Mesh] OR "Health Care Costs"[Mesh]) AND ("Dementia"[Mesh] OR "Frontotemporal Dementia"[Mesh] OR "Dementia, Multi-Infarct"[Mesh] OR "Dementia, Vascular"[Mesh] OR "Alzheimer Disease"[Mesh] OR "Lewy Body Disease"[Mesh] OR Dementia OR Alzheimer Disease OR Alzheimer's OR Alzheimer's Disease OR Lewy Body Disease OR MCI OR "Mild cognitive impairment")

Filters: Publication date from 2010/01/01-2016/12/31

EconLit

TX (dementia OR frontotemporal dementia OR Alzheimer Disease OR "Lewy Body Disease" OR MCI OR "mild cognitive impairment" OR vascular dementia OR Alzheimer's Disease) AND (TX (Cost OR costs OR economic evidence OR cost-utility OR cost-effectiveness OR cost-benefit OR saving))

Boolean/Phrase - TX TX (dementia OR frontotemporal dementia OR Alzheimer Disease ...

Expanders - Apply related words; Also search within the full text of the articles; Apply equivalent subjects

Limiters - Published Date: 20100101-20161231

Centre for Reviews and Dissemination

((MeSH DESCRIPTOR dementia EXPLODE 2) OR (((dementia or "frontotemporal dementia" or Alzheimer or "Lewy Body Disease" or MCI or "mild cognitive impairment" or "vascular dementia" or "Alzheimer''s Disease") AND (Cost or costs or economic evidence or cost-utility or cost-effectiveness or cost-benefit or saving)))) and ((Systematic review:ZDT and Bibliographic:ZPS) OR (Systematic review:ZDT and Abstract:ZPS) OR (Cochrane review:ZDT) OR (Cochrane related review record:ZDT) OR (Economic evaluation:ZDT and Bibliographic:ZPS) OR (Economic evaluation:ZDT and Abstract:ZPS) OR Project record:ZDT OR Full publication record:ZDT) IN DARE, NHSEED, HTA FROM 2010 TO 2016

Search results

Database	Number of references
PubMed	3303
EMBASE	4434
The Cochrane Library<br>▪ Trials<br>▪ Technology Assessments<br>▪ Economic Evaluations	326<br>(273)<br>(11)<br>(42)
PsycINFO	1116
EconLit	599
Centre for Reviews and Dissemination	269
**Total**	**10047**
