# Supplementary material for: Health economic evaluations of non-pharmacological interventions for persons with dementia and their informal caregivers: a systematic review
Source: BMC Geriatr. 2018 Mar 9;18:69. doi: 10.1186/s12877-018-0751-1 (PMC5845149; doi:10.1186/s12877-018-0751-1)
Supplement: Supplementary file 2 — Quality assessment of included studies according to the 10-item Drummond check-list. (PDF 199 kb) [file 12877_2018_751_MOESM2_ESM.pdf]

Additional file 2: Quality assessment of included studies according to the 10-item Drummond check-list

**Check-list**
1. Was a well-defined question posed in answerable form?
2. Was a comprehensive description of the competing alternatives given (i.e. can you tell who did what to whom, where, and how often)?
3. Was the effectiveness of the programme or services established?
4. Were all the important and relevant costs and consequences for each alternative identified?
5. Were costs and consequences measured accurately in appropriate physical units (e.g. hours of nursing time, number of physician visits, lost work-days, gained life years)?
6. Were the cost and consequences valued credibly?
7. Were costs and consequences adjusted for differential timing?
8. Was an incremental analysis of costs and consequences of alternatives performed?
9. Was allowance made for uncertainty in the estimates of costs and consequences?
10. Did the presentation and discussion of study results include all issues of concern to users?

	**1**	**2**	**3**	**4**	**5**	**6**	**7**	**8**	**9**	**10**
D'Amico et al. (2015) [21]	Yes: Research objectives stated p.63. Perspectives clearly stated p.64.	Yes: Very brief description of intervention and care as usual presented p.64.	Yes: Effectiveness results briefly presented p.65. Reference to another paper.	Yes: Cost of delivering the program (capital and overhead costs) and health resource utilization included p.64.<br>Travel cost was only considered for those caregivers who requested travel refunds.<br>Informal caregiver time collected p.64.	Yes: Sources of unit cost descripted p.64-65.	Yes: National sources listed p.64. Costs reported in £, price year 2011.	For equipment that provides a benefit for more than a year, discount rate of 3.5 % was used p.64.	Yes: ICER & ICURs p.67.	Yes: Sensitivity analyses reported; Analyses from the societal perspective. p.68.	Yes: Results compared to other studies. Limitations and implications discussed p.68-69.

	1	2	3	4	5	6	7	8	9	10
D'Amico et al. (2016) [28]	Yes: Research objectives stated p.656. Perspectives clearly stated p.658.	Yes: Brief description of intervention given p.657. No description of the scope of treatment as usual.	Yes: Effectiveness results presented p.661.	Yes: Health & social service cost, and informal caregiver time collected p.659-661.	Yes: Sources of unit cost descripted p.658.	Yes: National sources listed p.658. Costs reported in £, price year 2011.	N/A: Follow up period of 12 weeks. For services and equipment that provide a benefit for more than a year, costs were annuitized at a discount rate of 3.5 % p.658.	Yes: ICER & ICURs p.663.	Yes: ICERs and 95 % CIs presented for different perspectives controlled for baseline socio-demographic variables. Cost-effectiveness acceptability curves (CEAC) are presented. p.662-.663.	Yes: Results compared to other studies. Discussion of limitations p.663-664.
Dahlrup et al. (2013) [30]	Yes: Research objectives stated p.182. Perspective not explicitly stated.	Yes: Detailed description of intervention p. 184-186. No description of the scope of care as usual.	Yes: Effectiveness results presented p.188-190.	Cannot tell: Data gathered on home help service, adult day care service and specific nursing home placement p.186.<br><br>Costs for the meeting premises and private costs of the caregiver were not considered.	Yes: Data on resource use obtained from municipality registers and self-reports from family caregivers p.186.	Yes: Local tariffs were used to value the resource use. All nominal tariffs and costs for the intervention were indexed to 2010 price levels p.186.	Yes p.186.	No.	No: No sensitivity analysis reported.	Yes: Results compared to other studies. Discussion of limitations p.191-193
Davis et al. (2013) [33]	Yes: Research objectives stated p.2 Perspective clearly stated p.2.	Yes: Description of interventions and comparator reported p.3. Referred to separate publication for a more detailed description.	Yes: Very brief description of effectiveness results presented p.4.	Yes: Cost of delivering the program (capital and overhead costs) and health resource utilization included p.3 & 6.	Cannot tell: healthcare resource utilization questionnaire was self-complete by person with probable MCI may be subject to recall bias. Unit cost for each component of resource utilization reported p.5.	Yes: National sources listed p.3. Costs reported in CAD$, price year 2011.	N/A: Follow up period of 6 months.	Yes: ICER p.6.	Yes: Nested imputation and nonparametric bootstrapping were used to model uncertainty around the estimates for costs and effectiveness p.4-7.	Cannot tell: Results compared to another study. No discussion on generalizability. Recommendation for future research given. p.4-8.
Gitlin et al. (2010) [23]	Yes: Research objectives clearly stated p.511. Perspective clearly stated (individual caregiver) p.512.	Yes: Description of interventions and comparator reported p.511-512. Referred to separate publication for a more detailed description.	Yes: Description of effectiveness results reported p.514.	Yes: cost categories presented (cost of the intervention, caregiver time, travel time and expenses) p.512-513.	Yes: Cost calculation assumptions reported p. 513.	Cannot tell.	N/A: Follow up period of 4 months.	Yes: ICER p.515-516.	Yes: Sensitivity analyses reported p.516-517.	Yes: Results compared to other studies. Discussion of limitations p.517-518.

	1	2	3	4	5	6	7	8	9	10
Van Houtven et al. (2012) [32]	Yes: Research objective stated p.60. Perspective stated p.61.	Yes: Description of intervention and comparator reported p.62.	Cannot tell: Details on outcome measures stated in a referenced article p. 62. Only broad information stated on effectiveness of the intervention.	Cannot tell: 17 cost categories stated in detail p.62-63. Solely considering care-related out-of-pocket expenditures. Indirect cost of caregiving (transportation), expenditures for over the counter drugs was not included.	Cannot tell: Expenditures were assessed directly.	N/A.	N/A: Follow up period of 12 months.	No.	Yes: Sensitivity analyses conducted p.63-64. (Differential effects by caregiver relationship and by disease type of the care recipient reported.)	Yes: Results were compared to other studies and the generalizability was discussed. Study limitations were described p. 66-69.
Joling et al. (2013) [29]	Yes: Research objectives stated p.2. Perspective clearly stated p.2.	Yes: Description of intervention and comparator reported p.3. Full details given in separate paper.	Yes: RCT. Brief description of effectiveness results p.7. Full details given in separate paper.	Yes: Direct and indirect costs of both the caregiver and patient were gathered p.3-4. However, travel and capital costs were not included.	Yes: Cost breakdown p.4.	Yes: National sources listed p.3-4. Costs reported in EUR, price year 2009.	N/A: Follow up period of 1 year.	Yes: ICER & ICUR reported p.7-9.	Yes: 95 % CI and CEAC presented. Sensitivity analyses conducted:<br>(1) complete case analysis<br>(2) Analysis without an adjustment for baseline imbalances between IG and IC.	Yes: Results compared to other studies. Discussion of limitations and generalizability. Brief recommendation for future research given. p.10-12.
Laakkonen et al. (2016) [24]	Yes: Research objectives stated p.753. Perspective not explicitly stated (use and costs of health and social services)	Yes: Detailed description of intervention and comparator (care as usual) p. 754.	Yes: Description of effectiveness results p.755-756.	Yes: Resource usage information of health and social services retrieved from central registers and medical records for 2 years after randomization or until the participant's death p. 753.	Yes: Unit cost and resource use were clearly reported p.758.	Yes: National sources listed p.753-754. Costs reported in EUR, price year 2006.	Cannot tell.	Cannot tell: incremental costs per person per year p.757.	Yes: 95 % CI presented for between-group comparisons of cost and outcome data.	Yes: Results compared to other studies. Discussion of limitations and generalizability p.757-759.
Livingston et al. (2014) [18]	Yes: Research objective stated p.539. Perspective explicitly reported p.539.	Yes: Intervention and comparator were descripted in detail p.540.	Yes: Detailed presentation of effectiveness results p.543-544.	Yes: Costs were calculated separately for carers' and patients' service use. Informal care was not included p.541.	Yes:<br>Cost for the months 13 to 21 were estimated by interpolation p. 541.	Yes: The base year for unit costs was 2009-10 p. 541.	Yes: Discount rate of 3.5 % applied to costs and outcome p.541.	Yes: ICERs presented p.544-545.	Yes: Sensitivity analyses conducted (baseline imbalances, missing data). Non-pragmatic bootstrapping employed to estimate 95 % CIs for mean costs.	Yes: Results compared to other studies. Discussion of limitations p.546-547.

	1	2	3	4	5	6	7	8	9	10
Livingston et al. (2014) [19]	Yes: Research objectives stated p.5. Perspective explicitly stated p.19.	Yes: Intervention and comparator were descripted in detail p.8-14.	Yes. Detailed presentation of clinical outcomes.	Cannot tell: costs of treatment and care services used by the PwD was not included p.50.	Yes: Unit cost of services presented in detail p.65-66.	Yes: National sources listed p.19. The base year for unit costs was 2009-10 p.19.	Yes: Discount rate of 3.5 % applied to costs and outcome p.20.	Yes: ICERs presented p.33-34.	Yes: Sensitivity analyses conducted (baseline imbalances, missing data). Variation of discounting rate conducted p.20. CEACs and net monetary benefit.	Yes: Discussion of limitations and generalizability.
Orgeta et al. (2015) [27]	Yes: Research objectives clearly stated p.2. Perspectives explicitly stated p.20.	Yes: Intervention (and its development) and comparator were descripted in detail p.3-14.	Yes: RCT. Effectiveness results presented in detail p.32-38.	Yes: Intervention costs, service use by PwDs. Informal care cost, caregiver's lost employment and out-of-pocket costs.	Yes: Unit cost stated for utilized services & the intervention p.22-23.	Yes: National sources listed. The base year for unit costs was 2012–13 p.12–13.	N/A: Follow up period of 26 weeks.	Yes: ICER presented from different perspectives p. 48.	Yes: Sensitivity analyses conducted. Different approaches were employed to value informal care p.49-50. CEACs were generated p.93-94.	Yes: Implications and generalizability discussed and recommendations for future research presented in detail p.61-62.
Pitkälä et al. (2013) [20]	Yes: Research objectives stated p.895. Perspective not explicitly stated, apparently a health & social care perspective was used.	Yes: Intervention and comparator were descripted in detail p. 895-896.	Yes: RCT p.894. Effectiveness results presented in detail p.897-898.	Yes: Total health care and social care cost for patients & caregivers and cost of the intervention.	Cannot tell: Unit cost and resource use was clearly reported. Cost breakdown p.899. Breakdown of intervention cost not reported.	Yes: Costs were initially calculated in EUR and then changed to US$. Service costs based on unit costs according to registries in 2006 were inflated to 2012 values.	No.	No.	Yes: SD or 95 % CI were indicated for outcomes. Bootstrap estimations were used to derive 95 % CIs for the cost data.	Yes: Results compared to other studies. Discussion of limitations and generalizability p.898-900.
Søgaard et al. (2014) [22]	Yes: Research objectives stated p.1. Perspective clearly stated p.2.	Yes: Description of intervention and comparator reported p.2. Full details given in separate paper.	Yes: RCT. EQ-5D outcome values not presented. Mean QALY per group presented p.5.	Yes: cost of the intervention, informal caregiver time costed. Costing of health care based on national registries for service utilization p. 2.	Yes: Micro-costing of the intervention cost reported in a referred paper p.2.	Yes: National sources listed p.2. Costs reported in EUR, price year 2008.	Yes: Both costs and QALY were discounted at an annual rate of 3 % p.3.	No.	Yes: Alternative scenario analysis conducted p. 4-5. CEAC presented for alternative analytical scenarios p. 7.	Yes: Results compared to other studies. Discussion of limitations and practical implications p.7.

	1	2	3	4	5	6	7	8	9	10
Spector et al. (2015) [25]	Yes: Research objectives stated p.509. Perspective clearly stated p.511.	Yes: Detailed description of intervention and controls reported p.510-511.	Yes: RCT: Effectiveness results presented p.513-514.	Yes: cost of the intervention was included p.513-514.	Yes: information on health and social care services, equipment or adaptations, medication and accommodation p.510.	Yes: Unit costs were valued according to national sources p.511.	N/A: Follow up period of 6 months.	No.	Yes: Sensitivity analyses conducted (1. No imputation of missing cost data, 2. Exclusion of cost outliners) p. 514. SD or 95 % CI were indicated for outcomes. Bootstrap estimations were used to derive 95 % CIs for the cost data.	Yes: Limitations and implications discussed and recommendations for future research presented in detail p.51-54.
Woods et al. (2012) [26]	Yes: Research objectives clearly stated p.3. Perspective clearly stated p.18.	Yes: Detailed description of intervention and comparator reported p.9-10.	Yes: RCT. Effectiveness results presented in detail p.28-29.	Yes: cost of the intervention was included p.37-38.	Yes: Micro-costing of the intervention reported p.37-38. Cost breakdown of health care, social care and voluntary sector service use and associated costs by participants with dementia and their caregivers p.39-41	Yes: National sources listed p.18. The price year used was 2010 p.41.	N/A: Follow up period of 10 months.	Yes: ICER p.44.	Yes: CEACs planned, however not generated as the intervention is not cost-effective p.43. Cost-effectiveness planes were generated. 95 % CI was reported for the ICER.	Yes: Limitations and implications discussed and recommendations for future research presented in detail p.51-54.
Wray et al. (2010) [31]	Yes: Research objectives stated p.624. Perspective not explicitly stated (health care and social service perspective).	Yes: Detailed description of intervention and comparator reported p.624-626.	No: Details on outcome measures stated in a referenced article p. 624. Only broad information stated on the effectiveness of the intervention.	Cannot tell: Cost of the intervention was not included.	Yes: Health care cost: inpatient, nursing home, outpatient and outpatient pharmacy cost. Medicare cost data was not available for the follow-up periods.<br><br>Utilization data comprised: inpatient bed days, contract hospital files, long-term care files, and outpatient visits p.630.	No: No pricing year reported, no details given on potential price adjustments for inflation.	N/A: Follow up period of 1 year.	No.	Yes: Separate analyses, comparison of participants who received any additional treatments with the ones who do not p.626-628.	Yes: Results compared to other studies. Practical implications and generalizability discussed p.629-631.
